# Supplementary material for: Altered gene expression profiles impair the nervous system development in individuals with 15q13.3 microdeletion
Source: Sci Rep. 2022 Aug 5;12:13507. doi: 10.1038/s41598-022-17604-2 (PMC9356015; doi:10.1038/s41598-022-17604-2)
Supplement: Supplementary file 1 — Supplementary Information 1. [file 41598_2022_17604_MOESM1_ESM.pdf]

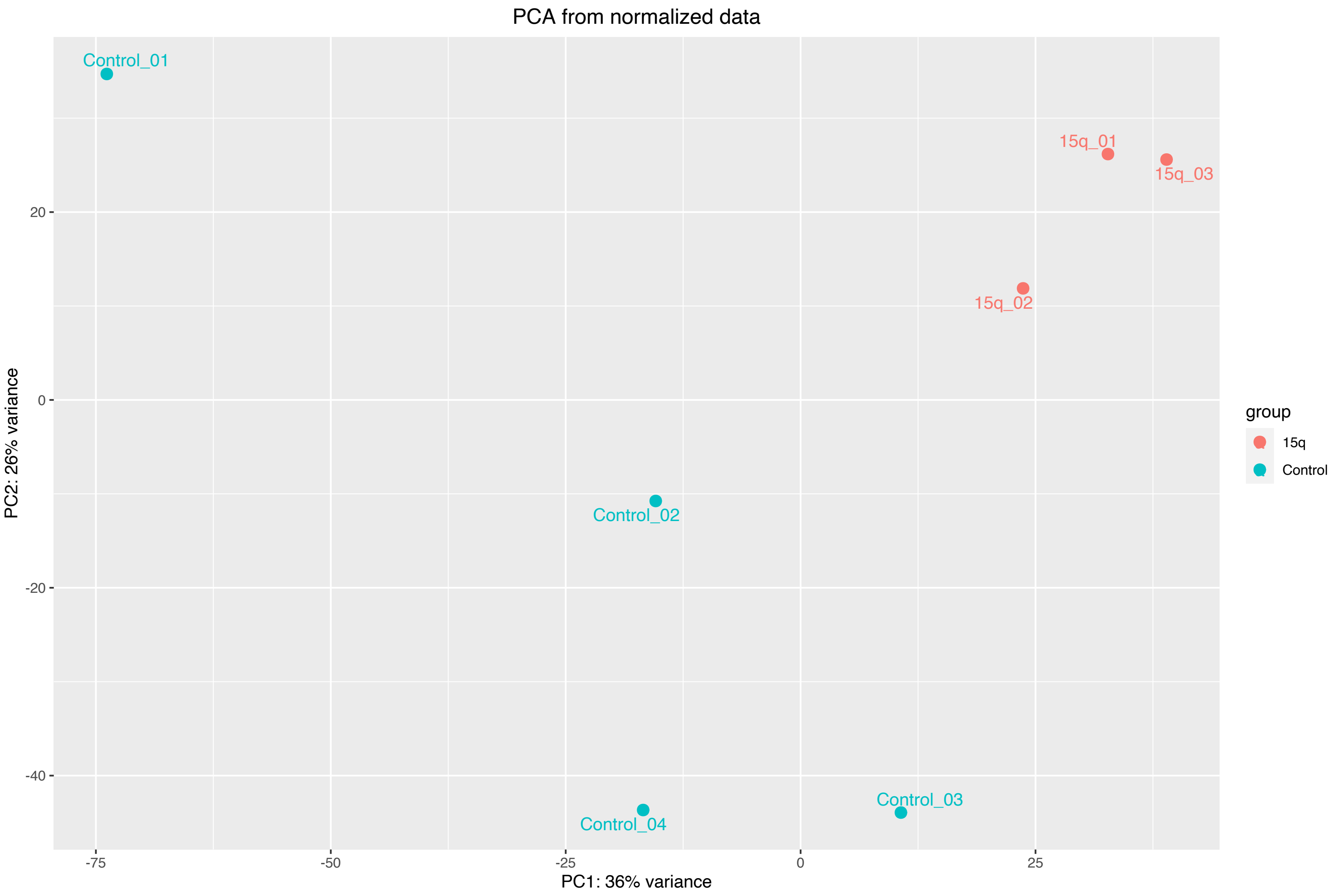

Aditional file 1. Principal component analysis (PCA) plot of human RNA-seq samples. The first two principal components (PCs) are plotted and coloured according to group (15q – individuals with 15q13.3 microdeletion syndrome and Control – age- and gender-matched controls). PCA was performed using all expressed genes. Percentage of variation accounted for by each principal component is shown with the axis label.
