# Supplementary material for: Altered gene expression profiles impair the nervous system development in individuals with 15q13.3 microdeletion
Source: Sci Rep. 2022 Aug 5;12:13507. doi: 10.1038/s41598-022-17604-2 (PMC9356015; doi:10.1038/s41598-022-17604-2)
Supplement: Supplementary file 3 — Supplementary Information 3. [file 41598_2022_17604_MOESM3_ESM.pdf]

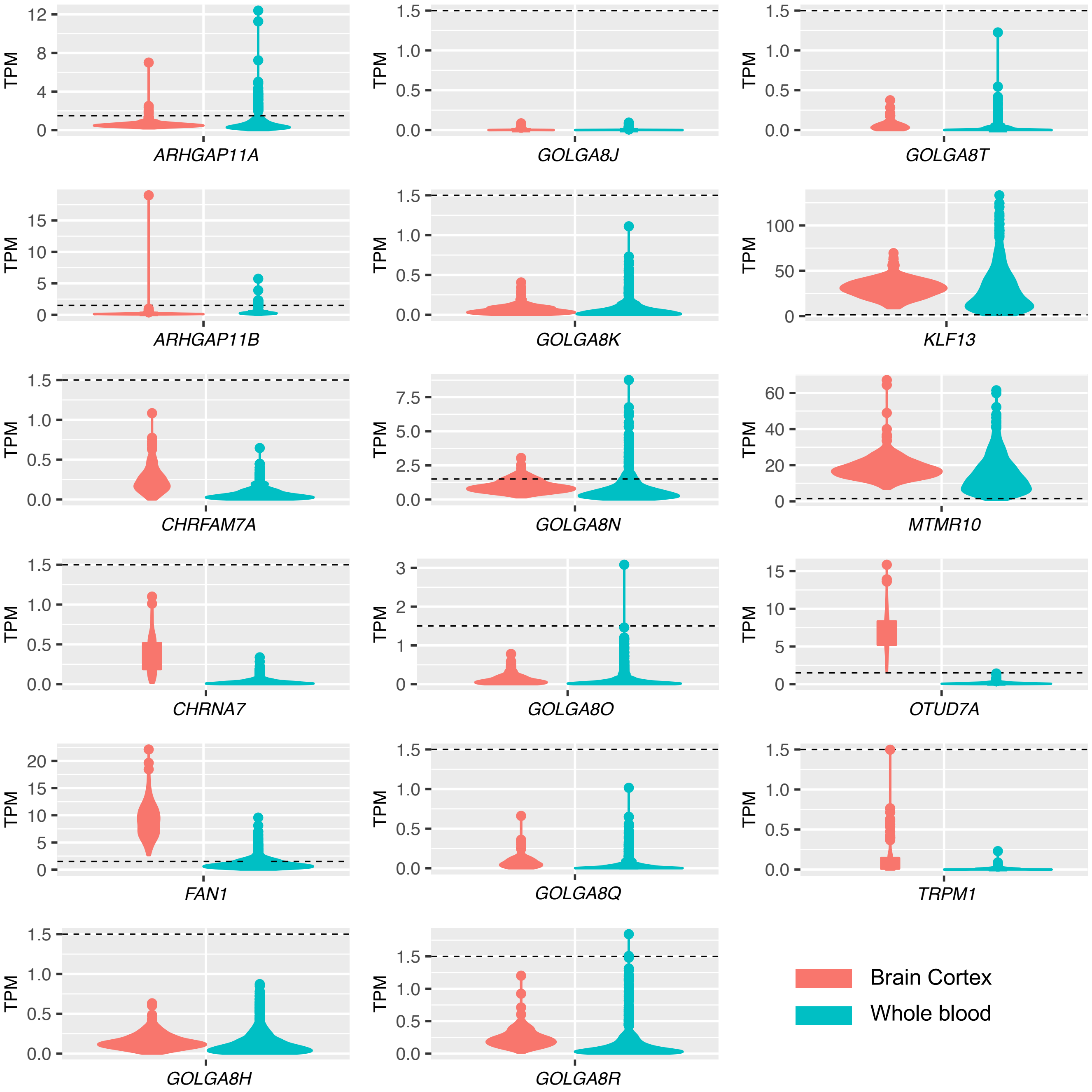

Additional file 3. Expression levels of genes located in the 15q13.3 microdeletion region. TPM (transcript per kilobase million mapped reads) values of gene expression levels are depicted for brain cortex tissue and whole blood. The dashed lines represent 1.5 TPM. Expression values were obtained from PTEE (https://bioinf.eva.mpg.de/PTEE/).
