# Supplementary material for: Altered gene expression profiles impair the nervous system development in individuals with 15q13.3 microdeletion
Source: Sci Rep. 2022 Aug 5;12:13507. doi: 10.1038/s41598-022-17604-2 (PMC9356015; doi:10.1038/s41598-022-17604-2)
Supplement: Supplementary file 6 — Supplementary Information 6. [file 41598_2022_17604_MOESM6_ESM.pdf]

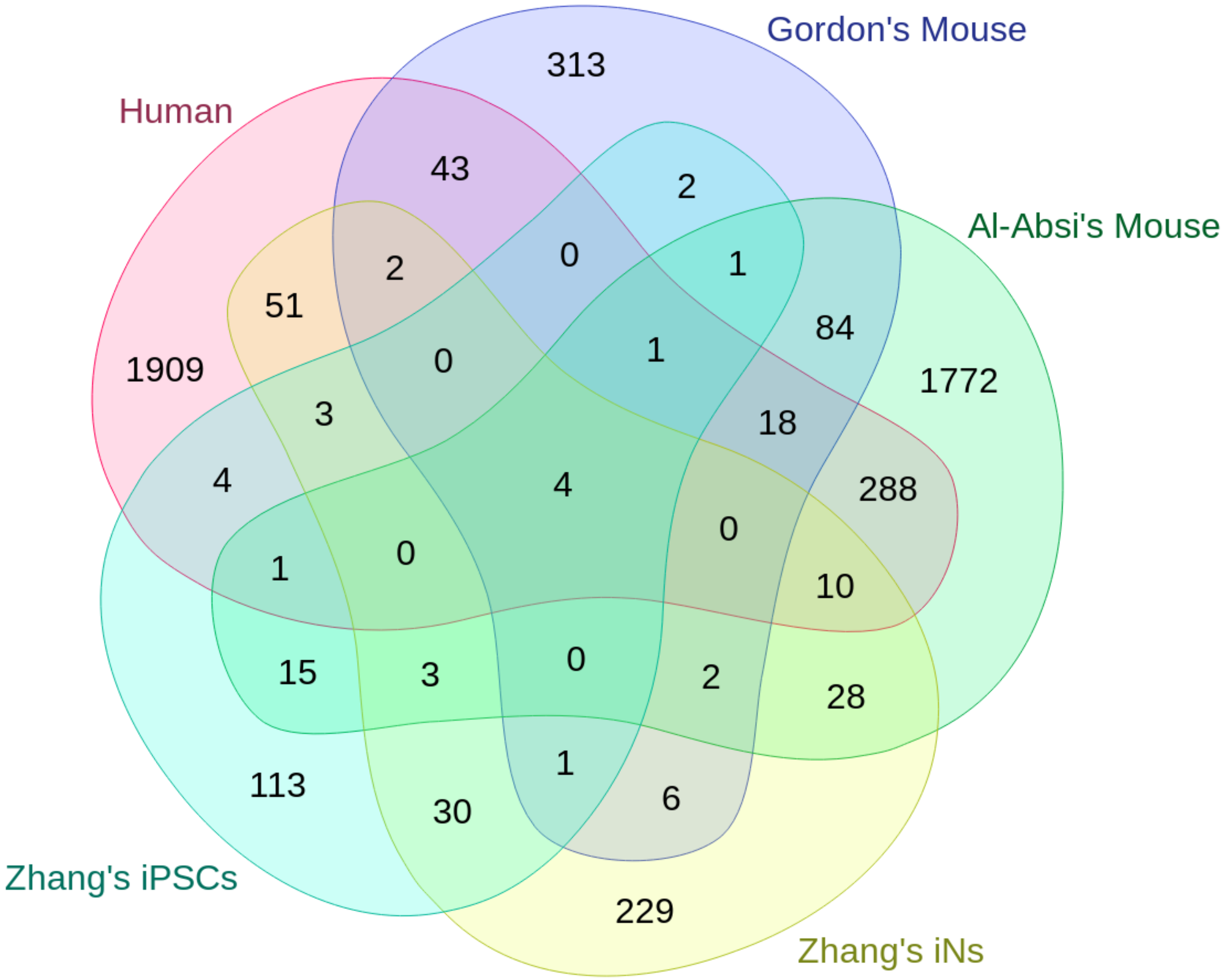

Additional file 6. Overlap between differentially expressed genes from our study on human blood RNA-seq of 15q13.3 microdeletion subjects (Human), mouse (Df[h15q13]/+) cerebral cortex reported by Gordon *et al*. 2021 (Gordon's Mouse), mouse (Df[h15q13]/+) medial prefrontal cortex reported by Al-Absi *et al*. 2021 (Al-Absi's Mouse), and induced pluripotent stem cells (Zhang's iPSCs), as well as induced neurons (Zhang's iNs) from Zhang *et al*. 2021. There is a significant overlap between the human blood RNA-seq and differentially expressed mouse genes data (p-value < 0.001) and the human blood RNA-seq data and induced neurons (p-value < 0.001). However, the overlap between the iPSCs and iNs and the two mouse models is not significant.
